# Long-Term Risk of Residual or Recurrent CIN 2–3 After LLETZ in Immunosuppressed vs. Immunocompetent Women: A 20-Year Cohort Study

**DOI:** 10.3390/cancers18111695

**Published:** 2026-05-22

**Authors:** Christian Leonardo Molina-Hinojosa, Ramón Carreras-Collado, María Saumoy-Linares, Judith Peñafiel, Fatima Heydari, Joan Climent Martí, María Eulalia Fernández-Montolí

**Affiliations:** 1 Doctoral Program in Pediatrics, Obstetrics and Gynecology, Doctoral School, Universitat Autònoma de Barcelona, 08193 Bellaterra, Barcelona, Spain; 1552538@uab.cat; 2 Department of Gynecology, Hospital del Mar, Universitat Autònoma de Barcelona, 08193 Bellaterra, Barcelona, Spain; rcarreras@parcdesalutmar.cat; 3 HIV and STI Unit, Department of Infectious Diseases, Bellvitge University Hospital, 08907 L’Hospitalet de Llobregat, Barcelona, Spain; msaumoy@bellvitgehospital.cat; 4 Biostatistics Unit, Bellvitge Biomedical Research Institute (IDIBELL), 08908 L’Hospitalet de Llobregat, Barcelona, Spain; jpenafiel@idibell.cat; 5 Medicine and Translational Research Doctorate Program, Faculty of Medicine and Health Sciences, Universitat de Barcelona, 08907 L’Hospitalet de Llobregat, Barcelona, Spain; fheydahe7@alumnes.ub.edu; 6 Department of Immunology, Germans Trias i Pujol Research Institute (IGTP), Hospital Universitari Germans Trias i Pujol, 08916 Badalona, Barcelona, Spain; jcliment.germanstrias@gencat.cat; 7 Department of Gynecology, Bellvitge University Hospital, Biomedical Research Institute (IDIBELL), Universitat de Barcelona, 08907 L’Hospitalet de Llobregat, Barcelona, Spain

**Keywords:** cervical intraepithelial neoplasia grade 2–3, immunosuppression, HIV, LLETZ, recurrence, associated factors, surgical margins, HPV persistence, long-term follow-up, immunovirological profile

## Abstract

**Background**: Immunosuppressed women are at increased risk of residual or recurrent high-grade cervical intraepithelial neoplasia (CIN 2–3) after excisional treatment, yet long-term comparative data remain limited. Previous studies are often small and heterogeneous, and they rarely compare outcomes directly with immunocompetent populations. This study evaluated the long-term incidence, timing and associated factors of CIN 2–3 recurrence after large loop excision of the transformation zone (LLETZ), stratified by immune status. **Methods**: We conducted a retrospective cohort study including 283 women treated with LLETZ for CIN 2–3 between 1996 and 2016 at Bellvitge University Hospital in Barcelona, Spain. Of these, 41 were immunosuppressed and 242 immunocompetent. Clinical, histopathological, virological, and immunological variables were extracted from hospital and pathology registries. Kaplan–Meier estimates and Cox proportional hazards models adjusted for immunosuppression status were used to evaluate time-to-recurrence and factors associated with recurrence. **Results**: At 36 months post-treatment, the probability of residual/recurrent CIN 2–3 was 44% in immunosuppressed women versus 5% in immunocompetent women (HR = 10.42, 95% CI 4.70–23.08, *p* < 0.001). Recurrence appeared earlier in immunosuppressed women (median 7 vs. 13 months). Persistent high-risk HPV infection at first follow-up (HR = 23.6, 95% CI 5.44–102, *p* < 0.001) and positive surgical margins (HR = 3.88, 95% CI 1.45–10.3, *p* = 0.007) were among the factors most strongly associated with recurrence, and advanced immunodeficiency (CD4+ < 200 cells/mm^3^ or detectable HIV viral load) was associated with earlier recurrences, though this association was not maintained after accounting for immunosuppression status in Cox models. **Conclusions**: Immunosuppressed women are at significantly higher and earlier risk of residual/recurrent CIN 2–3 after LLETZ. These findings support a risk-adapted, multidisciplinary follow-up integrating gynecologic, infectious disease, and immunologic care. Tailored surveillance and perioperative HPV vaccination may enhance secondary prevention in this high-risk population.

## Simple Summary

Persistent or recurrent cervical precancerous lesions can develop even after surgical treatment. Women with weakened immune systems, particularly those living with HIV, are at especially high risk. In this study, we retrospectively analyzed the long-term outcomes of immunosuppressed and immunocompetent women treated for high-grade cervical intraepithelial neoplasia at a university hospital in Barcelona, Spain. The objective was to determine whether immune status is associated with differences in risk, timing and related factors of residual or recurrent disease over time. We found that 34.1% of immunosuppressed women developed residual or recurrent CIN 2–3 compared with 4.6% of immunocompetent women and that recurrences appeared earlier in immunosuppressed women (median 7 vs. 13 months). Persistent high-risk HPV infection and positive surgical margins were among the factors most strongly associated with recurrence, while exploratory analyses suggested that lower CD4+ counts and detectable HIV viral load were associated with earlier recurrences. These findings provide real-world evidence that immune dysfunction not only increases recurrence risk but also compresses the window in which disease reappears. Integrating gynecologic, infectious disease, and immunologic follow-up may improve outcomes. More personalized surveillance strategies could help reduce the burden of cervical cancer in women with compromised immunity.

## 1. Introduction

Despite being one of the most preventable malignancies, cervical cancer remains a major global health challenge. It ranks as the eighth most common cancer and the ninth leading cause of cancer-related death among women worldwide, accounting for more than 300,000 deaths annually, despite the widespread availability of screening and HPV vaccination programmes [1,2,3]. While incidence and mortality have markedly declined in high-income regions such as Western Europe and the United States owing to widespread implementation of these programmes [4,5,6,7,8], cervical cancer remains disproportionately prevalent in low- and middle-income countries, where limited access to screening, diagnosis, and treatment services amplifies the burden [3,9]. In Spain, both incidence and mortality have declined in recent decades [10,11,12]. However, residual risk now concentrates primarily among women with insufficient screening coverage, and among immunocompromised populations, particularly those living with HIV [11,13,14,15,16]. While some reports have noted a higher incidence in younger women, this pattern remains inconsistent across studies and appears largely related to differences in sexual behaviour and HPV exposure, rather than age itself [10,17,18]. This reflects a global convergence of two epidemics, HIV and cervical cancer, particularly evident in sub-Saharan Africa [19]. The WHO’s 90–70–90 strategy underscores this reality, aiming to eliminate cervical cancer through universal HPV vaccination, high-coverage screening [20], and timely treatment, while emphasizing the urgent need to reach women left behind by prevention [19].

The natural history of human papillomavirus (HPV) infection underlies the pathogenesis of cervical precancer and invasive carcinoma [21]. Although most HPV infections are transient and spontaneously cleared within two years [22], persistent infection with oncogenic HPV genotypes may lead to the development of high-grade cervical intraepithelial neoplasia (CIN 2–3) and, ultimately, invasive cancer [23]. More than 200 HPV genotypes have been identified, at least 15 of which are considered high-risk [24,25]. Among these, HPV16 and HPV18 account for approximately 70% of cervical cancers worldwide [26,27,28,29,30,31]. In immunocompetent women, the progression from initial infection to precancer and invasive disease typically occurs over 10–20 years, providing a wide preventive and treatment window [24]. In contrast, immunosuppressed women—including those living with HIV, transplant recipients, and patients on long-term immunosuppressive therapy—experience a distinct clinical course [32,33,34,35,36,37]. They are more frequently infected with non-16/18 high-risk HPV types (e.g., HPV52, HPV58) [29,38,39,40], often present with multiple concurrent infections, and experience reduced clearance rates [41,42]. This biological interplay between HPV persistence and impaired immune surveillance results in accelerated progression from infection to high-grade lesions and invasive cancer [9,43], with a shorter interval to invasive disease of approximately 3.2 years compared with 15.7 years in the general population [40]. Furthermore, cervical precancer in immunosuppressed women shows a higher propensity for persistence or recurrence after excisional treatment, complicating long-term management [44,45,46,47,48].

Beyond host and viral factors [49], the characteristics of the surgical specimen—such as excision type, margin status [50], cone volume and geometric dimensions (length, thickness, circumference)—have also been linked to residual disease and recurrence [51,52,53]. According to the IFCPC classification [54], excisional treatments are categorized into three types based on the transformation zone (TZ) involved, reflecting the extent of endocervical involvement and surgical complexity.

These recommendations, endorsed by the ESGO, EFC, IFCPC, ESP [55], and AEPCC [56], as well as the NHS [57], provide standardized surgical benchmarks to balance oncologic safety and reproductive preservation [58,59]. Such parameters facilitate cross-study comparability and outcome reproducibility. However, despite these well-defined surgical standards, most available data originate from immunocompetent populations, and whether excision type, specimen dimensions, and margin status maintain equivalent prognostic value in immunosuppressed women remains uncertain.

Despite clear biological and clinical distinctions, current management guidelines continue to recommend similar treatment approaches for immunosuppressed and immunocompetent women [6,60]. However, emerging evidence suggests that standard protocols likely underestimate the risk of residual or recurrent high-grade lesions in immunosuppressed patients [61]. In this context, clarifying the true incidence, timing, and associated factors of recurrence in this vulnerable population is essential to guide tailored surveillance strategies.

The present study aimed to evaluate the long-term incidence, timing, and associated factors of residual or recurrent CIN 2–3 after large loop excision of the transformation zone (LLETZ), comparing immunosuppressed and immunocompetent women. Our findings can provide real-world evidence on how immune status is associated with recurrence risk, with potential implications for personalized post-treatment follow-up protocols and preventive strategies in high-risk women.

## 2. Materials and Methods

### 2.1. Study Design and Setting

We conducted a retrospective cohort study at Bellvitge University Hospital, a tertiary referral center in Barcelona, Spain. Participants were included over a 20-year period (1996–2016). For the present analysis, follow-up time was calculated from the date of LLETZ to the first occurrence of residual or recurrent CIN 2–3, hysterectomy, loss to follow-up, or last documented normal visit, whichever came first.

### 2.2. Participants

Women referred to the Department of Gynecology between January 1996 and December 2006 from primary care gynecology units (ASSIR) or other hospitals in the catchment area were screened for eligibility.

Eligible participants were adult women (≥18 years), immunocompetent or immunosuppressed, treated with LLETZ for histologically confirmed CIN 2–3, and had at least one post-treatment follow-up visit.

Exclusion criteria included histological diagnoses other than CIN 2–3 in the excised specimen (e.g., CIN 1 or invasive carcinoma), lack of follow-up, immediate retreatment after LLETZ (re-excision or hysterectomy), unknown immune status, or incomplete clinical data. Loss to follow-up was attributed to missed appointments, relocation, or death.

### 2.3. Objective

To evaluate the long-term probability of recurrence and timing of residual or recurrent CIN 2–3 following LLETZ, comparing immunosuppressed and immunocompetent women, and to identify clinical, surgical, virological, and immunological factors associated with recurrence in both groups.

### 2.4. Procedures and Data Collection

All LLETZ procedures were performed under standardized local anesthesia protocols. Excision depth reflected transformation zone (TZ) type; when indicated (TZ type 3 or age > 35), a second smaller endocervical “top-hat” pass was added. Surgical specimens were oriented at the time of excision, inked, and processed in the pathology department, where excision type, surgical margin status, number of quadrants affected, glandular extension, specimen dimensions and volume were recorded. Colposcopic findings and cytology results were initially documented according to the standards and classifications in use at the time of care (including earlier IFCPC and Bethesda systems) [62,63]. To ensure consistency across the two-decade study period, all reports were retrospectively reviewed and recoded following the IFCPC 2011 nomenclature and the Bethesda system (initially published in 1989 and updated in 1991, 2001, and 2014) [64]. Excision type was classified following National Health Service (NHS) standards [57]. HPV testing was performed using Hybrid Capture 2 (HC2; Qiagen, Hilden, Germany) during the earlier years of the study period and the Cobas 4800 PCR assay (Roche Molecular Systems, Pleasanton, CA, USA), thereafter, following implementation in routine clinical practice [65,66]. Semi-quantitative viral load estimation using relative light units (RLU) was only applicable during the period when HC2 testing was used, as PCR-based assays such as Cobas do not provide a direct measure of viral load. HPV viral load was categorized using a 100 relative light unit (RLU) cut-off. In women living with HIV, plasma HIV viral load and CD4 T-cell counts and percentages were extracted from the HIV clinic records closest to the date of LLETZ. For descriptive purposes, HIV RNA and CD4 values were summarized both in their original units and, when highly skewed, after natural-logarithmic transformation (Appendix A Table A1). In the survival models, log-transformed HIV RNA and CD4 values were used as continuous covariates to improve model fit and approximate linearity, whereas clinically interpretable categories (e.g., undetectable, low-level viremia, virological failure; CDC CD4 stages) were used to aid clinical interpretation. According to the current U.S. Department of Health and Human Services (HHS) guidance [67], an HIV RNA below the assay limit of detection (typically <20 copies/mL) was considered virologic suppression; low-level viremia was defined as confirmed HIV RNA above the limit of detection but <200 copies/mL; and virologic failure as persistent HIV RNA ≥ 200 copies/mL despite antiretroviral therapy [68]. CD4 counts were interpreted according to CDC staging criteria (stage 1: ≥500 cells/mm^3^ or ≥26%, stage 2: 200–499 cells/mm^3^ or 14–25%, stage 3: <200 cells/mm^3^ or <14%, or any AIDS-defining condition) [69]. Immunosuppression was considered based on clinically established conditions, including patients with HIV infection, solid organ transplantation, autoimmune diseases or hematologic disorders receiving chronic immunosuppressive therapy. For women living with HIV, objective and standardized immunological parameters were available (CD4 T-cell count and plasma HIV RNA), interpreted according to CDC staging criteria.

For non-HIV causes of immunosuppression, no widely adopted or standardized quantitative scoring system is currently used across clinical practice or epidemiological research [70]. Therefore, immunosuppression was treated as a categorical variable (immunosuppressed vs. immunocompetent), consistent with prior literature and real-world clinical classification.

Data were abstracted from medical records and cross-verified with hospital archives, and infectious disease and pathology registries to minimize errors and missing information.

### 2.5. Type of Excision, Specimen Measurements, and Volume Estimation

Excision type and cone morphology were assessed according to the 2011 IFCPC terminology [54] and NHS 2016 guidelines [57]. Type 1 corresponds to a completely ectocervical TZ, generally requiring a shallow resection of 7–10 mm. Type 2 targets a partially endocervical but fully visible TZ, with a resection depth of 10–15 mm, whereas Type 3 involves a deeper resection (15–25 mm) for lesions extending into the endocervical canal or associated with glandular or microinvasive features. These parameters defined the target depth and geometry of excision used for morphometric assessment.

Immediately after LLETZ, each specimen was opened, pinned on cork, and measured before formalin fixation to minimize shrinkage. Three linear dimensions were recorded following IFCPC [54] and WHO criteria [71]:

(i) Length, the distance from the ectocervical to the endocervical margin (depth of excision);
(ii) Thickness, the stromal depth from the base to the epithelial surface;
(iii) Circumference, the external perimeter of the opened specimen, reflecting horizontal extension.

When multiple fragments were obtained, measurements were taken separately and summed to derive total dimensions.

Cone volume was calculated using two validated geometric models, both based on the hemi-ellipsoid approximation of the excised specimen.

The Phadnis model [72] first conceptualized the specimen the cone as a hemi-ellipsoid (Equation (1)):

(1) $$V=\frac{1}{2}\times \frac{4}{3}\times \pi \times \frac{a}{2}\times \frac{b}{2}\times c$$

where *a* = transverse diameter, *b* = longitudinal diameter, and *c* = depth.

The Carcopino model [73] subsequently reformulated this approach using IFCPC-defined parameters (Equation (2)).

(2) $$V=\frac{1}{2}\times \frac{4}{3}\times \pi \times Length\times \left(\frac{Circumference}{2\pi }\right)\times Thickness$$

expressing volume as a function of specimen length, circumference, and thickness. As shown in Equation (2), this model achieved the highest concordance with true volumetric measurements obtained by fluid displacement (intraclass correlation coefficient = 0.47, 95% CI 0.36–0.56) [73].

All volumes were reported in cm^3^. These standardized definitions, consistent with recent Bellvitge series [51,53], enable reproducibility and cross-study comparability in evaluating excision geometry and recurrence risk.

### 2.6. Post-Treatment Follow-Up

Postoperative surveillance consisted of cytology and colposcopy at 6 and 12 months, with high-risk HPV testing at 6 months, according to national guidelines [74,75]. In cases with positive surgical margins or when abnormalities were detected, the first follow-up was advanced to 3 months. Patients were routinely provided with standard postoperative recommendations, including temporary avoidance of vaginal intercourse to allow adequate cervical healing and reduce the risk of infection or reinfection.

Residual disease was defined as histologically confirmed CIN 2–3 within 12 months after LLETZ, and recurrent disease as CIN 2–3 detected thereafter. HPV viral load is generally defined as the quantity of viral DNA present in a clinical sample. In the present study, viral load was not directly quantified using a fully quantitative molecular assay but was approximated using semi-quantitative signal intensity (relative light units, RLU) obtained from the Hybrid Capture 2 assay. This approach was applicable only during the period when HC2 testing was used.

Women without events were censored at last contact or study end; the time scale was measured in months from LLETZ.

### 2.7. Outcomes

The primary outcome was histologically confirmed CIN 2–3 detected after excisional treatment, analyzed as probability and time to first event.

Secondary outcomes included the association between clinical (age, parity, smoking), surgical (excision type, margin status and location, number of quadrants affected), virological (HPV persistence and viral load), and immunological factors (CD4 count, plasma HIV viral load) with the risk of residual or recurrent disease.

### 2.8. Statistical Analysis

Multiple imputation by chained equations was used to handle missing data [76,77], and was applied exclusively to the variable “type of treatment”, which had 16 missing values prior to imputation. Ten imputed datasets were generated and combined using Rubin’s rules. The assumption of missing at random was considered appropriate, as no systematic pattern of missingness was identified [78].

Categorical variables are presented as counts and percentages, and continuous variables as medians with interquartile range (IQR) and compared between immunosuppression groups using the chi-square test or Fisher’s exact test for categorical variables, and the Wilcoxon rank-sum test for continuous variables, as appropriate.

Time-to-recurrence was analysed using Kaplan–Meier estimates and compared between immunosuppressed and immunocompetent women using the log-rank test. Univariable Cox proportional hazards models were used to assess factors associated with recurrence. Each clinically relevant variable identified in univariable analyses was subsequently evaluated in a separate Cox model adjusted for immunosuppression.

All time-to-event analyses were administratively censored at 192 months.

The proportional hazards assumption was formally assessed using Schoenfeld residuals, and no relevant violations were detected. All tests were two-sided, with *p* < 0.05 considered statistically significant. Analyses were performed using R (version 4.0.2; R Foundation for Statistical Computing, Vienna, Austria).

### 2.9. Ethical Approval

This study was conducted within the framework of a doctoral research project at the Universitat Autònoma de Barcelona (UAB) and involved a retrospective observational analysis of previously collected anonymized clinical data. According to the Research Ethics Committee of the Universitat Autònoma de Barcelona (CERec), this study did not require formal ethics clearance, as confirmed in an official institutional statement.

The study was conducted in accordance with the principles of the Declaration of Helsinki (2013 revision) and complied with the European General Data Protection Regulation (GDPR; EU 2016/679) and Spanish Organic Law 3/2018 on the protection of personal data. No identifiable patient information was accessed at any stage of the study.

The data supporting the findings of this study are available from the corresponding author upon reasonable request, subject to applicable privacy and data protection regulations.

## 3. Results

### 3.1. Study Population

**Figure 1:**
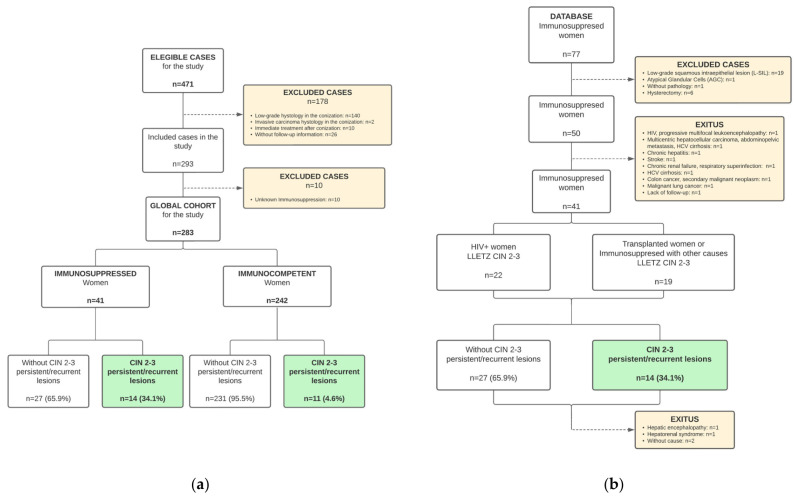
Participant selection flowchart: (**a**) Selection of the overall study cohort. (**b**) Selection of the subset of immunosuppressed women included in the analysis. Green boxes indicate persistent/recurrent CIN 2–3 lesions, whereas beige boxes indicate excluded cases or deaths.

A total of 471 women treated with LLETZ for histologically confirmed CIN 2–3 between 1996 and 2016 were initially identified. After applying the exclusion criteria—including CIN 1, invasive carcinoma, immediate retreatment (re-excision or hysterectomy), missing follow-up data, unknown immune status, or incomplete clinical information—283 women were eligible, including 41 (14.5%) immunosuppressed and 242 (85.5%) immunocompetent women. Patients were included over a 20-year period (1996–2016). This timeframe reflects the study inclusion period rather than the duration of follow-up. Follow-up varied across participants, with a median follow-up of 28 months (IQR 8–139). Among immunosuppressed women, 22 (53.7%) were HIV-positive and 19 (46.3%) had other causes of immunosuppression, including renal transplantation, autoimmune diseases, haematologic disorders, or long-term systemic immunotherapy (Figure 1).

Outcomes in immunocompetent women from this cohort have been comprehensively characterised elsewhere (Fernández-Montolí et al., BJOG 2019 [52]; Medina-Gonzalo et al., Life 2024 [51]) and are briefly summarised here as a benchmark for comparison. The present study focuses on immunosuppressed women, highlighting their distinct clinical course and recurrence dynamics compared with immunocompetent women.

### 3.2. Descriptive Statistics

#### 3.2.1. Demographic and Immunological Data

The median age at treatment was 37.5 (IQR 31.2–45.1 years) in immunosuppressed women, slightly higher than that reported in immunocompetent women from the same cohort [51,52]. Nearly half of the patients were younger than 35 years at the time of LLETZ. Parity and smoking status distributions were similar across excision types, with no statistically significant differences (*p* > 0.05) (Table A1).

Among the 41 immunosuppressed women who underwent LLETZ, most had good virological control at the time of treatment. Median HIV RNA was 0.0 copies/mL (IQR 0–99), consistent with virologic suppression or low-level viremia according to current HHS criteria. However, HIV RNA levels differed significantly according to excision type (*p* = 0.020): women treated with type 1 LLETZ had consistently undetectable HIV RNA (0.0 (0.0–0.0) copies/mL), whereas those requiring type 2 excisions showed a broader range of values, including several cases within the virological failure range 99.0 (0.0–4039) copies/mL, implying that at least one quarter of these women had HIV RNA ≥ 200 copies/mL. Women treated with type 3 excisions showed intermediate levels, typically within the spectrum of virologic suppression or low-level viremia, 49.5 (0.0–99.0) copies/mL (Table A1).

Regarding immunological status, CD4-related variables were assessed exclusively in women living with HIV. Overall, CD4 counts and percentages covered a broad range, from levels compatible with severe immunosuppression (stage-3 range < 200 cells/mm^3^ or <14%) to partially reconstituted immunity (stage 2 range 200–499 cells/mm^3^ or 14–25%). Among women with available data, upper quartiles for CD4 count and CD4 percentage fell within 300–480 cells/µL and 17–28%, but the skewed distribution and the high proportion of missing values precluded a reliable estimate of how many women fell into each CDC immunologic stage. No statistically significant differences in CD4 counts or percentages were observed across LLETZ types (*p* > 0.05) (Table A1).

Baseline characteristics of immunocompetent women have been reported in detail elsewhere; a summary is provided here for reference (Table 1).

#### 3.2.2. Surgical Specimen Findings

Positive surgical margins were more frequent in immunosuppressed than in immunocompetent women (58.5% vs. 44.6%), although this difference did not reach statistical significance (*p* = 0.19) [51,52]. Among positive margins, isolated ectocervical involvement (ecto+/endocervix−) was the most common pattern in both groups (19.5% vs. 17.4%), followed by isolated endocervical involvement (ecto−/endocervix+; 12.2% vs. 9.1%) and combined ectocervical and endocervical involvement (ecto+/endocervix+; 7.3% vs. 2.5%). Glandular involvement was present in 29.3% of immunosuppressed and 33.1% of immunocompetent women.

The extent of disease differed significantly between groups (*p* < 0.001). In immunosuppressed women, four quadrants were involved in 48.1% of cases and three quadrants in 18.5%, compared with 22.3% and 8.7% of immunocompetent women, respectively. Involvement of one or two quadrants was similar or slightly less common in immunosuppressed than in immunocompetent women (11.1% vs. 15.3% and 18.5% vs. 20.7%, respectively).

Specimen morphometry and excision characteristics were also analysed. Median cone circumference was 5.34 (IQR 4.40–6.28 cm) in immunosuppressed women and 5.34 (IQR 4.71–6.28 cm) in immunocompetent women (*p* = 0.469). Median cone thickness was 1.00 (IQR 0.88–1.15 cm) and 1.05 (IQR 0.92–1.25 cm), respectively (*p* = 0.380). Median cone length was 1.10 (IQR 0.78–1.50 cm) in immunosuppressed women and 0.90 (IQR 0.68–1.20 cm) in immunocompetent women (*p* = 0.151). Median cone volume estimated by the Carcopino formula was 1.81 (1.25–2.26) cm^3^ in immunosuppressed women and 1.88 (1.16–2.79) cm^3^ in immunocompetent women (*p* = 0.724), and by the Phadnis formula 0.58 (0.40–0.72) vs. 0.60 (0.37–0.89) cm^3^ (*p* = 0.727), with no statistically significant differences in any of these morphometric parameters between groups. Type 1 excision was performed in 39.0% of immunosuppressed women and 45.0% of immunocompetent women, type 2 in 31.7% versus 34.3%, and type 3 in 29.3% versus 20.7%, with no statistically significant differences in the distribution of excision types between groups (*p* = 0.462). Overall, these findings show the broader lesion extent among immunosuppressed women compared with immunocompetent counterparts, despite comparable excision parameters (Table 1).

#### 3.2.3. HPV Detection and Viral Load

At first follow-up, overall HPV detection after LLETZ was more frequent in immunosuppressed than in immunocompetent women (30.8% vs. 15.5%; *p* = 0.015). High-risk HPV positivity at first follow-up was also significantly higher in immunosuppressed women compared with immunocompetent counterparts (47.5% vs. 17.4%, *p* < 0.001). Median HPV viral load was also significantly higher in immunosuppressed women than in immunocompetent patients (2.00 RLU vs. 0.26 RLU, *p* < 0.001). When viral load was categorised, values >100 RLU at first follow-up were observed in 17.1% of immunosuppressed women and 4.55% of immunocompetent women (*p* = 0.001) (Table 1).

The prognostic impact of HR-HPV persistence and HPV viral load on residual/recurrent CIN 2–3 is detailed in the Cox regression analyses.

### 3.3. Incidence and Timing of Residual or Recurrent CIN 2–3

**Figure 2:**
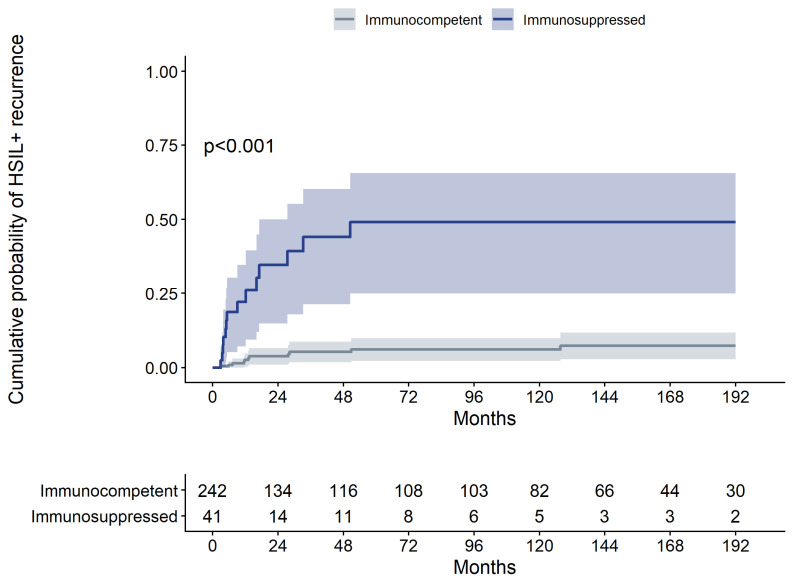
Kaplan–Meier: Time-to-recurrence according to immune status.

Residual or recurrent CIN 2–3 occurred in 14 of 41 immunosuppressed women (34.1%) and in 11 of 242 immunocompetent women (4.55%) (Table 2). In Kaplan–Meier analysis, at 36 months post-treatment, the probability of residual/recurrent CIN 2–3 reached 44% in the immunosuppressed group compared with 5% in the immunocompetent group (log-rank *p* < 0.001) (Figure 2).

The median time to residual or recurrent CIN 2–3 was significantly shorter in immunosuppressed women (7 months, IQR 4–17) than in immunocompetent women (13 months, IQR 9–28). Overall follow-up was longer in immunocompetent than in immunosuppressed women (median 30 (9–149) vs. 12 (5–51) months, respectively) (Table 2).

Patients without recurrence were censored at the last follow-up or at the predefined administrative censoring time (192 months), whichever occurred first, consistent with the time-to-event analysis framework.

### 3.4. Overview of Factors Evaluated for Recurrence

Potential associated factors of residual or recurrent CIN 2–3 were systematically evaluated across clinical, surgical, virological, and immunological domains. At the clinical level, women who developed recurrence were older, with those aged ≥35 years overrepresented in the recurrence group, whereas parity and smoking status did not differ significantly between women with and without recurrence. In contrast, several surgical parameters—including positive margins (particularly endocervical or combined involvement), multifocal high-grade disease tended to be more frequent among women who experienced recurrence, and larger cone dimensions—which may reflect more extensive baseline disease rather than associated factors—were also more frequent among women who experienced recurrence. Persistent high-risk HPV infection and high post-LLETZ HPV viral load levels (>100 RLU) were also more common in the recurrence group. Among immunosuppressed women, higher HIV viral loads at the time of LLETZ were associated with recurrence, whereas CD4 T-cell counts and CD4 percentages did not show a clear relationship with residual or recurrent disease. These trends were further explored through univariable analyses and Cox models adjusted for immunosuppression status to evaluate factors associated with recurrence.

### 3.5. Univariable Cox Regression in the Overall Cohort

In univariable Cox analysis of the overall cohort (Table 3), immunosuppression was strongly associated with residual or recurrent CIN 2–3 (HR = 10.4, 95% CI 4.70–23.1; *p* < 0.001). Age, when analysed as a continuous variable, was not significantly related to recurrence; however, women aged ≥ 35 years had a higher hazard than those <35 years (HR = 2.78, 95% CI 1.11–6.95; *p* = 0.029). Persistent high-risk HPV infection at the first follow-up visit was one of the strongest associated factors of recurrence (HR = 23.6, 95% CI 5.44–102; *p* < 0.001). An HPV viral load > 100 RLU at that visit conferred an even higher hazard (HR = 30.7, 95% CI 11.2–84.1; *p* < 0.001). Positive surgical margins were also significantly associated with recurrence (HR = 3.88, 95% CI 1.45–10.3; *p* = 0.007), with greater hazards when endocervical (HR = 5.97, 95% CI 1.82–19.6) or combined endo- and ectocervical margins were involved (HR = 8.78, 95% CI 1.77–43.6). These findings suggested that margin status and viral factors were variables selected for further evaluation in subsequent Cox models adjusted for immunosuppression. No significant associations were observed for type of excision, cone dimensions, or other covariates in univariable analyses.

### 3.6. Cox Models Adjusted for Immunosuppression for Factors Associated with Residual/Recurrent CIN 2–3

Factors identified in univariable analyses and those considered clinically relevant were entered into separate Cox proportional hazards models adjusted for immunosuppression (Table 4). High-risk HPV positivity at the first post-LLETZ visit remained associated with recurrence in models adjusted for immunosuppression (HR = 14.85, 95% CI 3.32–66.55; *p* < 0.001).

Age, entered as a continuous variable, was not significantly associated with recurrence after adjustment (HR = 1.02 per year, 95% CI 0.99–1.05; *p* = 0.272).

Treatment type was also not associated with recurrence after adjustment, either for type 2 versus type 1 excision (HR = 0.69, 95% CI 0.23–2.09; *p* = 0.939) or type 3 versus type 1 excision (HR = 0.47, 95% CI 0.10–2.31; *p* = 0.326). Cone dimensions and excision volume, estimated using the Carcopino and Phadnis formulas, were evaluated but did not show a significant association with recurrence.

Overall, these findings indicate that immunosuppression and persistent HR-HPV infection after treatment are the factors most consistently associated with residual or recurrent CIN 2–3, whereas treatment type and cone morphology did not appear to have a significant prognostic effect in this cohort.

### 3.7. Exploratory Analysis in Immunosuppressed Women

Whereas the main analyses focused on the entire cohort, additional exploratory analyses were performed within the predefined immunosuppressed subgroup (*n* = 41). Detailed baseline clinical, virological and immunological characteristics of these women, stratified by excision type, are shown in Appendix A (Table A1). Univariable Cox models for potential factors associated with residual or recurrent CIN 2–3 in this subgroup are summarized in the Appendix B (Table A2). In these exploratory analyses, margin status and post-treatment HPV parameters were associated with recurrence. Compared with women with negative margins, those with isolated endocervical involvement showed an increased hazard of recurrence (HR = 6.06, 95% CI 1.27–28.9; *p* = 0.024), and those with combined endocervical and ectocervical involvement showed a higher hazard (HR = 14.0, 95% CI 2.11–92.4; *p* = 0.006). An initial post-LLETZ HPV viral load > 100 RLU was also associated with increased risk (HR = 5.54, 95% CI 1.45–21.1; *p* = 0.012). In contrast, differences in HIV RNA and CD4 values between women with and without recurrence were modest and, given the limited sample size, did not reach statistical significance in these exploratory models (Table A2).

Given the limited sample size (*n* = 41 immunosuppressed women and only 14 events), these immunovirological findings should be interpreted with caution due to the potential for unstable estimates and risk of bias inherent to subgroup analyses in retrospective studies and should be considered exploratory and hypothesis-generating rather than definitive. These results suggest that systemic immune status (HIV infection and CD4 depletion) and local factors (margin status and HPV persistence) may interact to shape individual risk, but further studies with larger samples are needed to confirm these associations.

## 4. Discussion

Persistent or recurrent CIN2–3 after excisional treatment represents a major clinical challenge, particularly in women with impaired immunity. In this long-term cohort, we evaluate the burden, timing, and factors associated with recurrence following treatment.

In our study, immunosuppression markedly increases the frequency of residual or recurrent CIN2–3 after excisional treatment. At 36 months, the probability of recurrence reached 44% in immunosuppressed women compared with 5% in immunocompetent counterparts, corresponding to more than a tenfold increase in risk. These findings are consistent with prior reports in immunosuppressed populations, although recurrence rates have varied widely across studies [79,80,81,82,83,84,85,86,87,88,89,90,91,92,93,94]. Importantly, most prior series are limited by shorter follow-up or heterogeneous populations. In contrast, our study spans a 20-year inclusion period, enabling the capture of both early and late recurrences and offering a more comprehensive estimate of the long-term burden associated with immunosuppression.

Beyond the higher frequency of recurrence, events also occurred earlier among immunosuppressed women. The median time to recurrence was 7 months compared with 13 months in immunocompetent women, with more than half of the recurrences in immunosuppressed women occurring within the first year of follow-up, indicating a condensed recurrence window. This pattern is consistent with prior studies in women living with HIV, in which early persistence or recurrence of CIN2–3 within 6–12 months has been reported [95,96], while late recurrences can still arise several years after treatment [92,97]. In most series of immunocompetent populations treated with LLETZ, recurrences occur within the first 24 months after treatment [52,95,98,99,100,101], but a smaller proportion diagnosed later, beyond 3–5 years [92,102].

The variability in timing reflects differences in immune status [103], antiretroviral therapy, excisional technique, and follow-up intensity [104,105]. Immunosuppression is known to weaken HPV clearance, favour multiple high-risk type co-infections [41], and alter the cervical microenvironment [41,103,106,107,108].

Overall, these findings suggest that, in the context of immunosuppression, residual or recurrent disease tends to emerge early, supporting the need for intensified surveillance during the first year after excision.

In addition to incidence and timing, age was associated with recurrence in univariable analyses, with women aged ≥ 35 years showing a higher risk compared with younger women. Older age likely reflects long-standing HPV exposure, cumulative cervical injury, and age-related changes in immune competence [52,109,110]. However, age did not retain statistical significance in Cox models after adjustment for immunosuppression, suggesting that the observed association may be influenced by other covariates, particularly immunosuppression and HPV-related factors. Accordingly, age should be interpreted as a risk modifier rather than a driver of recurrence [52,111,112,113,114,115,116,117,118]. Clinically, while age alone should not guide management decisions, women ≥ 35 years may benefit from closer follow-up, especially when additional associated factors coexist.

Regarding surgical factors, immunosuppressed women presented with more extensive baseline disease with involvement of three or four quadrants observed more frequently than in immunocompetent counterparts, reflecting a greater lesion burden at the time of LLETZ. However, quadrant involvement did not remain an associated factor of recurrence after adjustment, suggesting that its effect is mediated through other factors, particularly margin status. This contrasts with immunocompetent series in which involvement of ≥2–4 quadrants predicted residual/recurrent disease, where greater circumferential involvement correlated with endocervical margin positivity [119,120,121,122,123,124]. In this context, more extensive disease likely increases the probability of incomplete excision and residual microscopic disease rather than acting as a direct driver of recurrence.

Consistent with this, our data reinforce the central role of margin status. Women with any margin involvement had substantially higher recurrence, particularly when the endocervical margin or both margins were affected. This finding mirrors large series and meta-analyses showing that involved margins, especially endocervical, are among the strongest associated factors of residual disease after LLETZ [50,51,84,125,126,127,128,129,130]. The stronger effect observed in immunosuppressed women suggests that incomplete excision may act synergistically with impaired local immune defense, facilitating multifocal, residual or newly developing high-grade lesions over time [131,132,133].

After adjustment for post-treatment HPV status and margin involvement—the main associated factors of recurrence—no association was observed between excision type, cone dimensions or calculated cone volume and recurrence. Although cone length was greater in immunosuppressed women and associated with endocervical margin clearance, it behaved as an intermediate variable and lost significance after adjustment. These findings suggest that the apparent benefit of larger or deeper excisions is primarily mediated by increased likelihood of negative margins rather than cone size per se [51,53]. At the same time, more extensive excisions have been consistently associated with increased obstetric morbidity without a clear reduction in treatment failure [55,58,134,135,136], with this risk being more pronounced in cold-knife conization and laser techniques, while generally lower with LLETZ.

Cone thickness showed no association with margin status or recurrence in our cohort, consistent with prior reports indicating that only very thin excisions are associated with an increased margin positivity [55]. Cone circumference was likewise unrelated to recurrence, in contrast to studies in immunocompetent women where circumferential involvement of ≥50% of the ectocervix has been linked to residual disease and margin positivity [137]. Similarly, calculated cone volume, assessed using both the Carcopino and Phadnis formulas, did not demonstrate an association with recurrence in Cox models adjusted for immunosuppression, whereas in immunocompetent women, cone volumes < 2 cm^3^ have been associated with positive margins [138]. Overall, these findings suggest that, in immunosuppressed women, cone dimensions act mainly as technical descriptors of excision rather than oncologic determinants, with recurrence driven predominantly by margin status and post-treatment HPV persistence.

Regarding the type of excisional procedure, most women in our global cohort underwent a type 1 excision. However, the mean cone length corresponded more closely to type 2 excisions, suggesting some degree of mismatch between the recorded excision type and the actual cone dimensions in routine practice [139,140]. In our series, immunosuppressed women required type 3 excisions more than immunocompetent women, reflecting the higher prevalence of extensive lesions and TZ3 in this group. However, excision type was not associated with residual or recurrent CIN2–3, suggesting that applying excisional algorithms derived from immunocompetent populations is insufficient to overcome an adverse biological background. These findings contrast with some series in immunocompetent women, where more extensive excisions targeting TZ3 have been associated with lower failure [51,141]. Overall, these data support a more individualized surgical strategy aimed at securing clear margins and incorporating immune status, HPV persistence and lesion characteristics, rather than relying solely on TZ-based excision categories while avoiding unnecessarily large excisions, balancing oncological safety against reproductive harm [142].

Virological markers were among the strongest associated factors of recurrence in our cohort. A positive high-risk HPV test at the first post-LLETZ visit was associated with a markedly increased risk of recurrence, while high viral load (>100 RLU) identified an even higher-risk subgroup. Numerous systematic reviews and meta-analyses have shown that post-treatment HPV testing is more sensitive than cytology and at least as informative as margin status for detecting residual or recurrent CIN2+ [143,144,145]. Moreover, higher HPV viral load was associated with increased risk of CIN2+ during follow-up [109,146,147]. These parameters reflect ongoing viral replication, which—when combined with systemic immunosuppression—creates a favourable setting for disease reactivation.

Immunological status showed a consistent association across all models. Immunosuppressed women had a markedly higher risk of residual/recurrent CIN2–3 compared with immunocompetent women, even after adjustment within Cox models accounting for immunosuppression status. Although the magnitude of effect observed in our cohort was greater than that reported in prior studies, the direction of association is consistent, reinforcing impaired immune function as a key determinant of treatment failure [47,48,148].

Within our cohort, most immunosuppressed patients exhibited CD4 values ranging from severe immunosuppression to partially reconstituted immunity [69,149]. Women with lower CD4+ counts showed a tendency toward earlier and more frequent recurrences, supporting the role of cellular immunity in controlling high-risk HPV infection. Conversely, immune reconstitution under effective antiretroviral therapy was associated with longer disease-free intervals, in line with evidence that sustained viral suppression and CD4+ recovery reduce recurrence risk [150,151,152].

Beyond systemic immunosuppression, molecular interactions between HIV and HPV may further accelerate cervical carcinogenesis. HIV-related immune dysfunction impairs HPV clearance, while viral proteins and chronic inflammation may enhance HPV transcription and persistence [40,153]. In immunosuppressed women, the predominance of non-16/18 high-risk HPV genotypes has also been associated with increased persistence and genomic integration, potentially contributing to the heterogeneous recurrence patterns observed [40]. These mechanisms support a model of a self-reinforcing pattern of viral persistence and impaired immune control, in which immune exhaustion and sustained HPV infection interact to promote earlier recurrence, consistent with the accelerated kinetics observed in our cohort.

Although most women in our cohort had suppressed HIV viral load, a subset presented residual viremia at the time of treatment. While our study was underpowered to assess its effect, persistent viremia likely reflects incomplete immune restoration and may contribute to recurrence risk [67,154,155]. The observed discordance between immunologic and virologic responses further suggests that no single biomarker adequately captures recurrence risk in this population, supporting a more comprehensive assessment of immune status [156], viral control and adherence to ART (>2 years) [157]. These findings reinforce the potential role of integrated immunovirological monitoring to guide post-treatment surveillance strategies.

From a clinical perspective, our findings support a risk-adapted management approach in immunosuppressed women with CIN 2–3. Current guidelines, including those from ASCCP [6], NIH/CDC [60], AEPCC [18], Cancer Council Australia [158], and European Guidelines [159], generally apply similar treatment strategies to immunocompetent and immunosuppressed populations. However, the higher and earlier recurrence observed in our cohort suggests that intensified surveillance, particularly within the first year after treatment, may be warranted. HPV-based “test of cure” strategies remain central to follow-up [158], although in this high-risk group, they may require combination with cytology and colposcopy. In addition, perioperative HPV vaccination is emerging as a promising adjunct in secondary prevention, with ongoing studies expected to clarify its role [145,160].

This study has several limitations. Its single-center design may limit generalizability; however, it ensured homogeneous clinical management and centralized pathology assessment. The smaller number of immunosuppressed women reflects their lower representation in screening programs and the use of strict inclusion criteria, resulting in wider confidence intervals. Subgroup analyses within the immunosuppressed population may further introduce a risk of selection bias and residual confounding, given the limited number of events and the retrospective nature of the study and should therefore be interpreted as exploratory. Furthermore, immunosuppression represents a heterogeneous and multidimensional condition, and no standardized or widely validated quantitative scoring system is currently used for non-HIV immunosuppression [70]. Changes in diagnostic and follow-up practices over the study period, including HPV testing methods, may also have influenced detection; to mitigate this, analyses were based on clinically relevant categorizations and Cox models adjusted for immunosuppression and clinically relevant covariates, with results stable in sensitivity analyses restricted to time windows with uniform testing availability. HPV genotyping was not available for all cases, precluding genotype-specific analyses; however, post-treatment HR-HPV status and semi-quantitative signal intensity (RLU), when available, remained among the strongest prognostic markers. Finally, the retrospective design inherently carries potential for selection and information biases; we minimized these by cross-referencing independent data sources (hospital, pathology, and infectious disease registries) with rigorous data verification.

Despite these limitations, this study provides one of the most comprehensive long-term evaluations of post-excisional outcomes in immunosuppressed women, supported by extended follow-up, standardized histopathological confirmation, and integrated clinical, surgical, virological, and immunological data. A key strength is the multidisciplinary approach integrating gynecology, pathology, infectious disease and immunology expertise; and the use of rigorous statistical methodology, including imputation, to address limited missing data.

Future research should focus on validating these findings in multicenter cohorts and refining risk stratification through biomarker-driven approaches—including HPV genotyping, quantitative viral load, methylation markers (e.g., ASCL1, LHX8) [161], and vaginal microbiota profiling [82,162,163,164,165,166,167,168,169,170,171]. Interventional trials should evaluate integrated strategies combining optimized excision, structured HPV-based “test of cure” [42,172,173,174,175], and adjunctive vaccination in this high-risk population [176,177,178,179,180,181,182]. Emerging evidence suggests that HPV vaccination may extend beyond primary prevention, potentially enhancing viral clearance and reducing recurrence risk. Although these effects remain to be confirmed in immunosuppressed populations, they provide biological plausibility for incorporating vaccination into secondary prevention strategies. Such efforts could help redefine secondary prevention paradigms and reduce the disproportionate post-treatment burden borne by immunosuppressed women worldwide.

## 5. Conclusions

Immunosuppressed women are at a substantially higher and earlier risk of residual or recurrent CIN 2–3 after LLETZ compared with immunocompetent women. Recurrence is mainly driven by post-treatment high-risk HPV persistence and positive surgical margins, particularly when the endocervical or both margins are involved. In contrast, excision characteristics appear to play a secondary role compared with immunological and virological factors.

Accordingly, post-treatment management in immunosuppressed women should move toward risk-adapted surveillance strategies integrating gynecologic, virological, and immunological monitoring into a personalized follow-up framework.

Further prospective studies are warranted to validate these findings and to define the role of adjunctive strategies such as HPV vaccination and intensified surveillance in this high-risk population.

## Figures and Tables

**Table 1:** Demographic, clinical, surgical and HPV-related characteristics, including follow-up, according to immune status in women treated with LLETZ for CIN 2–3 at Bellvitge University Hospital, Barcelona, Spain (1996–2016).

	Total	Immunocompetent	Immunosuppressed	*p*-Value	*n*
	*n* = 283	*n* = 242	*n* = 41		
**Demographic characteristics of the patients**					
**Age (years)**					
Median (Q1; Q3)	36.0 (29.5; 44.0)	35.5 (29.3; 44.0)	37.5 (31.2; 45.1)	0.160	283
Age (Categorized at 35 years), *n* (%):				0.550	283
<35 years	133 (47.0%)	116 (47.9%)	17 (41.5%)		
35+ years	150 (53.0%)	126 (52.1%)	24 (58.5%)		
Parity, *n* (%):				0.214	283
Nulliparous	48 (17.0%)	41 (16.9%)	7 (17.1%)		
≤4 Full-term births	195 (68.9%)	170 (70.2%)	25 (61.0%)		
>4 Full-term births	16 (5.65%)	14 (5.79%)	2 (4.88%)		
Unknown	24 (8.48%)	17 (7.02%)	7 (17.1%)		
Parity (2), *n* (%):				0.110	283
Nulliparous or ≤4 Full-term births	243 (85.9%)	211 (87.2%)	32 (78.0%)		
>4 Full-term births	16 (5.65%)	14 (5.79%)	2 (4.88%)		
Unknown	24 (8.48%)	17 (7.02%)	7 (17.1%)		
Smokers, *n* (%):				0.128	283
No	159 (56.2%)	140 (57.9%)	19 (46.3%)		
Yes	98 (34.6%)	83 (34.3%)	15 (36.6%)		
Unknown	26 (9.19%)	19 (7.85%)	7 (17.1%)		
**Characteristics of the surgical specimen**					
**Involved surgical margins**					
Margin status, *n* (%):				0.19	283
Negative margins	151 (53.4%)	134 (55.4%)	17 (41.5%)		
Ecto+/endocervix−	50 (17.7%)	42 (17.4%)	8 (19.5%)		
Ecto−/endocervix+	27 (9.54%)	22 (9.09%)	5 (12.2%)		
Ecto+/endocervix+	9 (3.18%)	6 (2.48%)	3 (7.32%)		
All margins	7 (2.47%)	4 (1.65%)	3 (7.32%)		
Indeterminate	36 (12.7%)	31 (12.8%)	5 (12.2%)		
Unknown	2 (0.71%)	2 (0.83%)	0 (0.00%)		
Depth margin	1 (0.35%)	1 (0.41%)	0 (0.00%)		
Margin status (categorized), *n* (%):				0.251	283
Negative margins	151 (53.4%)	134 (55.4%)	17 (41.5%)		
Ecto+/−, Endo+/−, All, Depth	94 (33.2%)	75 (31.0%)	19 (46.3%)		
Indeterminate	36 (12.7%)	31 (12.8%)	5 (12.2%)		
Unknown	2 (0.71%)	2 (0.83%)	0 (0.00%)		
Glandular involvement, *n* (%):				0.366	283
No	15 (5.30%)	11 (4.55%)	4 (9.76%)		
Yes	92 (32.5%)	80 (33.1%)	12 (29.3%)		
Unknown	176 (62.2%)	151 (62.4%)	25 (61.0%)		
**Number of quadrants affected by H-SIL**					
Quadrants affected, *n* (%):				**<0.001**	269
0	1 (0.37%)	0 (0.00%)	1 (3.70%)		
1	40 (14.9%)	37 (15.3%)	3 (11.1%)		
2	55 (20.4%)	50 (20.7%)	5 (18.5%)		
3	26 (9.67%)	21 (8.68%)	5 (18.5%)		
4	67 (24.9%)	54 (22.3%)	13 (48.1%)		
Unknown	80 (29.7%)	80 (33.1%)	0 (0.00%)		
**Dimensions and volume of surgical specimen**					
Circumference *, Median (Q1; Q3)	5.34 (4.71; 6.28)	5.34 (4.71; 6.28)	5.34 (4.40; 6.28)	0.469	217
Thickness, Median (Q1; Q3)	1.05 (0.90; 1.25)	1.05 (0.92; 1.25)	1.00 (0.88; 1.15)	0.380	250
Volume LLETZ Carcopino **, Median (Q1; Q3)	1.85 (1.17; 2.67)	1.88 (1.16; 2.79)	1.81 (1.25; 2.26)	0.724	111
Volume LLETZ Phadnis ***, Median (Q1; Q3)	0.59 (0.37; 0.85)	0.60 (0.37; 0.89)	0.58 (0.40; 0.72)	0.727	111
Length, Median (Q1; Q3)	1.00 (0.70; 1.20)	0.90 (0.68; 1.20)	1.10 (0.78; 1.50)	0.151	127
Endocervical canal extension *, *n* (%)	79 (27.9%)	69 (28.5%)	10 (24.4%)	0.722	283
**Types of treatment**					
Type of treatment, *n* (%):				0.462	283
Excision type 1	125 (44.2%)	109 (45.0%)	16 (39.0%)		
Excision type 2	96 (33.9%)	83 (34.3%)	13 (31.7%)		
Excision type 3	62 (21.9%)	50 (20.7%)	12 (29.3%)		
**Data post LLETZ**					
Post-LLETZ HPV detection, *n* (%):				**0.015**	245
Negative	137 (55.9%)	129 (58.9%)	8 (30.8%)		
Positive	42 (17.1%)	34 (15.5%)	8 (30.8%)		
Unknown	66 (26.9%)	56 (25.6%)	10 (38.5%)		
**First HR-HPV post-LLETZ**, *n* (%):				**<0.001**	281
Negative	147 (52.3%)	137 (56.8%)	10 (25.0%)		
Positive	61 (21.7%)	42 (17.4%)	19 (47.5%)		
Unknown	73 (26.0%)	62 (25.7%)	11 (27.5%)		
**HPV Viral Load post-LLETZ**					
First HPV RLU post-LLETZ, Median (Q1; Q3)	0.28 (0.18; 1.47)	0.26 (0.17; 0.71)	2.00 (0.38; 422)	**<0.001**	191
First HPV RLU post-LLETZ (categorized), *n* (%):				**0.001**	283
Negative	140 (49.5%)	130 (53.7%)	10 (24.4%)		
1–100	33 (11.7%)	26 (10.7%)	7 (17.1%)		
>100	18 (6.36%)	11 (4.55%)	7 (17.1%)		
Not performed	92 (32.5%)	75 (31.0%)	17 (41.5%)		
**Follow-up**					
Months, Median (Q1; Q3)	27.5 (7.75; 139)	30.3 (8.48; 149)	11.9 (4.96; 50.5)	**0.001**	283

Abbreviations: HR-HPV, high-risk human papillomavirus; RLU, relative light units; ASC-US, atypical squamous cells of undetermined significance; HSIL, high-grade squamous intraepithelial lesion. Surgical specimen parameters were defined according to the 2011 colposcopic terminology of the International Federation of Cervical Pathology and Colposcopy (IFCPC) [54]. Bold values indicate statistically significant differences between groups. * Only in immunocompetent patients. ** *Carcopino* formula: $\frac{1}{2}\times \frac{4}{3}\times \pi \times Lenght\times \frac{Circumference}{2\pi }\times Thickness$ [73]. *** *Phadnis* formula: $\frac{1}{2}\times \frac{4}{3}\times \pi \times \frac{transverse\;diameter}{2}\times \frac{longitudinal\;diameter}{2}\times depth$ [72].

**Table 2:** Residual/recurrent cervical intraepithelial neoplasia grade 2–3 (CIN 2–3), time to residual/recurrent disease and follow-up according to immune status in women treated with large loop excision of the transformation zone (LLETZ) at Bellvitge University Hospital, Barcelona, Spain (1996–2016).

	Immunocompetent	Immunosuppressed	*n*
	*n* = 242	*n* = 41	
Residual/Recurrent Disease (CIN 2–3), *n* (%)	11 (4.55%)	14 (34.1%)	283
Months to Residual/Recurrent Disease (CIN 2–3), Median (Q1; Q3)	13.0 (9.36; 27.9)	7.18 (4.09; 16.9)	25
Follow-up (months), Median (Q1; Q3)	30.3 (8.48; 149)	11.9 (4.96; 50.5)	283

Abbreviations: CIN, Cervical intraepithelial neoplasia. Immunosuppressed women include those living with HIV or those with solid-organ transplantation/other causes of chronic immunosuppression. Residual/recurrent disease: CIN 2–3 lesions diagnosed after LLETZ excisional treatment, classified as residual if occurring within 12 months of follow-up, or recurrent if occurring after 12 months.

**Table 3:** Cox proportional hazards analysis of factors associated with residual/recurrent cervical intraepithelial neoplasia grade 2–3 (CIN 2–3), including immune status, in women treated with loop electrosurgical excision procedure (LLETZ) at Bellvitge University Hospital, Barcelona, Spain (1996–2016).

	Total	No Residual/Recurrent CIN 2–3	Residual/Recurrent CIN 2–3	HR	*p* Value	*n*
	*n* = 283	*n* = 258	*n* = 25			
Immunosuppression, *n* (%):						283
No	242 (85.5%)	231 (89.5%)	11 (44.0%)	Ref.	Ref.	
Yes	41 (14.5%)	27 (10.5%)	14 (56.0%)	10.4 (4.70; 23.1)	**<0.001**	
Age Median (Q1; Q3)	36.0 (29.5; 44.0)	35.3 (29.2; 43.8)	40.5 (36.0; 48.2)	1.03 (1.00; 1.06)	0.096	283
Categorized Age, *n* (%):						283
<35 years	133 (47.0%)	127 (49.2%)	6 (24.0%)	Ref.	Ref.	
35+ years	150 (53.0%)	131 (50.8%)	19 (76.0%)	2.78 (1.11; 6.95)	**0.029**	
Type of treatment, *n* (%):						283
Excision type 1, *n* (%)	125 (44.2%)	113 (43.8%)	12 (48.0%)	ref.	ref.	
Excision type 2, *n* (%)	96 (33.9%)	90 (34.9%)	6 (24.0%)	0.77 (0.27; 2.2)	0.599	
Excision type 3, *n* (%)	62 (21.9%)	55 (21.3%)	7 (28.0%)	0.56 (0.11; 2.84)	0.45	
First HR-HPV post-LLETZ, *n* (%):						281
Negative	147 (52.3%)	145 (56.4%)	2 (8.33%)	Ref.	Ref.	
Positive	61 (21.7%)	44 (17.1%)	17 (70.8%)	23.6 (5.44; 102)	**<0.001**	
Not performed	73 (26.0%)	68 (26.5%)	5 (20.8%)	4.72 (0.91; 24.3)	0.064	
Thickness Median (Q1; Q3)	1.05 (0.90; 1.25)	1.05 (0.90; 1.25)	1.12 (1.00; 1.25)	0.92 (0.26; 3.32)	0.902	250
Volume LLETZ Carcopino **, Median (Q1; Q3)	1.85 (1.17; 2.67)	1.87 (1.16; 2.79)	1.68 (1.41; 2.21)	0.78 (0.34; 1.75)	0.540	111
Volume LLETZ Phadnis ***, Median (Q1; Q3)	0.59 (0.37; 0.85)	0.59 (0.37; 0.89)	0.53 (0.45; 0.70)	0.45 (0.03; 5.82)	0.540	111
Margin status (categorized), *n* (%):						281
Negative margins	151 (53.7%)	145 (56.4%)	6 (25.0%)	Ref.	Ref.	
Ecto+/−, Endo+/−, All, Depth	94 (33.5%)	82 (31.9%)	12 (50.0%)	3.88 (1.45; 10.3)	**0.007**	
Indeterminate	36 (12.8%)	30 (11.7%)	6 (25.0%)	4.78 (1.54; 14.8)	**0.007**	
Margin status, *n* (%):						281
Negative margins	151 (53.7%)	145 (56.4%)	6 (25.0%)	Ref.	Ref.	
Ecto+/endocervical−	50 (17.8%)	46 (17.9%)	4 (16.7%)	2.19 (0.62; 7.78)	0.224	
Ecto−/endocervical+	27 (9.61%)	22 (8.56%)	5 (20.8%)	5.97 (1.82; 19.6)	**0.003**	
Ecto+/endocervical+	9 (3.20%)	7 (2.72%)	2 (8.33%)	8.78 (1.77; 43.6)	**0.008**	
All margins	7 (2.49%)	6 (2.33%)	1 (4.17%)	5.39 (0.64; 45.1)	0.120	
Indeterminate	36 (12.8%)	30 (11.7%)	6 (25.0%)	4.78 (1.54; 14.8)	**0.007**	
Depth margin	1 (0.36%)	1 (0.39%)	0 (0.00%)	0.00 (0.00;)	0.998	
Quadrants affected, *n* (%):						269
0–1	41 (15.2%)	39 (15.7%)	2 (9.52%)	Ref.	Ref.	
2	55 (20.4%)	51 (20.6%)	4 (19.0%)	1.30 (0.24; 7.12)	0.761	
3	26 (9.67%)	22 (8.87%)	4 (19.0%)	3.77 (0.69; 20.6)	0.126	
4	67 (24.9%)	59 (23.8%)	8 (38.1%)	2.62 (0.56; 12.3)	0.224	
Unknown	80 (29.7%)	77 (31.0%)	3 (14.3%)	0.83 (0.14; 4.97)	0.838	
First HPV RLU post-LLETZ, *n* (%):						283
0–100	173 (61.1%)	166 (64.3%)	7 (28.0%)	Ref.	Ref.	
>100	18 (6.36%)	8 (3.10%)	10 (40.0%)	30.7 (11.2; 84.1)	**<0.001**	
Unknown	92 (32.5%)	84 (32.6%)	8 (32.0%)	2.03 (0.73; 5.59)	0.173	

Abbreviations: HR, hazard ratio; HR-HPV, high-risk human papillomavirus; RLU, relative light units. Residual/recurrent disease: CIN 2–3 lesions diagnosed after LLETZ excisional treatment, classified as residual if occurring within 12 months of follow-up, or recurrent if occurring after 12 months. Immunosuppressed women include those living with HIV or those with solid-organ transplantation/other causes of chronic immunosuppression. Surgical specimen parameters were defined according to the 2011 International Federation of Cervical Pathology and Colposcopy (IFCPC) terminology [54]. Bold values indicate statistically significant differences. ** *Carcopino* formula: $\frac{1}{2}\times \frac{4}{3}\times \pi \times Lenght\times \frac{Circumference}{2\pi }\times Thickness$ [73]. *** *Phadnis* formula: $\frac{1}{2}\times \frac{4}{3}\times \pi \times \frac{transverse\;diameter}{2}\times \frac{longitudinal\;diameter}{2}\times depth$ [72].

**Table 4:** Bivariate Cox regression models adjusted for immunosuppression, evaluating each clinically relevant covariate associated with residual/recurrent cervical intraepithelial neoplasia grade 2–3 (CIN 2–3) in women treated with loop electrosurgical excision of the transformation zone (LLETZ) at Bellvitge University Hospital, Barcelona, Spain (1996–2016).

Factors	HR	CI	*p*
HR-HPV post-LLETZ (Positive vs. Negative)	14.85	3.32–66.55	**<0.001**
HR-HPV post-LLETZ (Not performed vs. Negative)	3.61	0.69–18.84	0.128
Age (years)	1.02	0.99–1.05	0.272
Treatment: Type 2 vs. Type 1	0.69	0.23–2.09	0.939
Treatment: Type 3 vs. Type 1	0.47	0.10–2.31	0.326

Abbreviations: HR, hazard ratio; HR-HPV, high-risk human papillomavirus; LLETZ, Large Loop Excision of the Transformation Zone. Bold values indicate statistically significant differences.

## Data Availability

Additional anonymized data and statistical outputs are available from the corresponding author upon reasonable request due to institutional policies and patient privacy restrictions. The doctoral thesis supporting this study [183] is publicly available at the TDX repository. Available online: http://hdl.handle.net/10803/694524, accessed on 29 April 2026.

## Abbreviations

The following abbreviations are used in this manuscript:

AEPCC	Asociación Española de Patología Cervical y Colposcopia
ASSIR	Atenció a la Salut Sexual i Reproductiva
CDC	Centers for Disease Control and Prevention
CI	Confidence Interval
CIN	Cervical Intraepithelial Neoplasia
EFC	European Federation for Colposcopy
ESGO	European Society of Gynaecological Oncology
ESP	European Society of Pathology
HIV	Human Immunodeficiency Virus
HPV	Human Papillomavirus
HR	Hazard Ratio
IFCPC	International Federation for Cervical Pathology and Colposcopy
LLETZ	Large Loop Excision of the Transformation Zone
NHS	National Health Service
RLU	Relative Light Units

## Appendix A

**Table A1:** Demographic, surgical specimen, HPV-related and follow-up characteristics of immunosuppressed women treated with large loop excision of the transformation zone (LLETZ) for CIN 2–3, according to excision type, at Bellvitge University Hospital, Barcelona, Spain (1996–2016).

	Total	Type 1	Type 2	Type 3	*p* Value	*n*
	*n* = 41	*n* = 16	*n* = 13	*n* = 12		
**Characteristics of Immunosupressed Patients**						
Age (years), Median (Q1; Q3)	37.5 (31.2; 45.1)	39.1 (33.9; 44.4)	36.3 (31.4; 42.6)	36.6 (30.5; 48.3)	0.731	41
Age (Categorized at 35 years), *n* (%):					0.572	41
<35	17 (41.5%)	5 (31.2%)	6 (46.2%)	6 (50.0%)		
35+	24 (58.5%)	11 (68.8%)	7 (53.8%)	6 (50.0%)		
Parity, *n* (%):					0.053	41
Nulliparous	7 (17.1%)	0 (0.00%)	5 (38.5%)	2 (16.7%)		
≤4 Full-term births	25 (61.0%)	12 (75.0%)	5 (38.5%)	8 (66.7%)		
>4 Full-term births	2 (4.88%)	0 (0.00%)	1 (7.69%)	1 (8.33%)		
Unknown	7 (17.1%)	4 (25.0%)	2 (15.4%)	1 (8.33%)		
Parity (2), *n* (%):					0.649	41
Nulliparous or ≤4 Full-term births	32 (78.0%)	12 (75.0%)	10 (76.9%)	10 (83.3%)		
>4 Full-term births	2 (4.88%)	0 (0.00%)	1 (7.69%)	1 (8.33%)		
Unknown	7 (17.1%)	4 (25.0%)	2 (15.4%)	1 (8.33%)		
Smokers, *n* (%):					0.903	41
No	19 (46.3%)	7 (43.8%)	6 (46.2%)	6 (50.0%)		
Yes	15 (36.6%)	5 (31.2%)	5 (38.5%)	5 (41.7%)		
Unknown	7 (17.1%)	4 (25.0%)	2 (15.4%)	1 (8.33%)		
**HPV Results and Cytology**						
Previous HPV, *n* (%):					0.100	41
Negative	1 (2.44%)	1 (6.25%)	0 (0.00%)	0 (0.00%)		
Positive	25 (61.0%)	9 (56.2%)	11 (84.6%)	5 (41.7%)		
Unknown	15 (36.6%)	6 (37.5%)	2 (15.4%)	7 (58.3%)		
Posterior HPV, *n* (%):					0.197	26
Negative	8 (30.8%)	3 (33.3%)	4 (50.0%)	1 (11.1%)		
Positive	8 (30.8%)	1 (11.1%)	3 (37.5%)	4 (44.4%)		
Not performed	10 (38.5%)	5 (55.6%)	1 (12.5%)	4 (44.4%)		
First HR-HPV post-LLETZ, *n* (%):					0.453	40
Negative	10 (25.0%)	4 (25.0%)	4 (33.3%)	2 (16.7%)		
Positive	19 (47.5%)	6 (37.5%)	7 (58.3%)	6 (50.0%)		
Not performed	11 (27.5%)	6 (37.5%)	1 (8.33%)	4 (33.3%)		
First HPV RLU post-LLETZ, Median (Q1; Q3)	2.00 (0.38; 422)	0.47 (0.24; 255)	1.74 (0.43; 335)	18.2 (1.48; 725)	0.469	24
First HPV RLU post-LLETZ (categorized), *n* (%):					0.100	41
Negative	10 (24.4%)	4 (25.0%)	4 (30.8%)	2 (16.7%)		
1–100	7 (17.1%)	0 (0.00%)	4 (30.8%)	3 (25.0%)		
>100	7 (17.1%)	2 (12.5%)	3 (23.1%)	2 (16.7%)		
Not performed	17 (41.5%)	10 (62.5%)	2 (15.4%)	5 (41.7%)		
Histology, *n* (%):					0.322	41
ASC-US	2 (4.88%)	2 (12.5%)	0 (0.00%)	0 (0.00%)		
HSIL	39 (95.1%)	14 (87.5%)	13 (100%)	12 (100%)		
**Characteristics of the surgical specimen**						
Margin status, *n* (%):					0.173	41
Negative margins	17 (41.5%)	7 (43.8%)	3 (23.1%)	7 (58.3%)		
Ecto+/endocervix−	8 (19.5%)	1 (6.25%)	4 (30.8%)	3 (25.0%)		
Ecto−/endocervix+	5 (12.2%)	2 (12.5%)	3 (23.1%)	0 (0.00%)		
Ecto+/endocervix+	3 (7.32%)	1 (6.25%)	0 (0.00%)	2 (16.7%)		
All margins	3 (7.32%)	2 (12.5%)	1 (7.69%)	0 (0.00%)		
Indeterminate	5 (12.2%)	3 (18.8%)	2 (15.4%)	0 (0.00%)		
Margin status (categorized), *n* (%):					0.299	41
Negative margins	17 (41.5%)	7 (43.8%)	3 (23.1%)	7 (58.3%)		
Ecto+/−, Endo+/−, All, Depth	19 (46.3%)	6 (37.5%)	8 (61.5%)	5 (41.7%)		
Indeterminate	5 (12.2%)	3 (18.8%)	2 (15.4%)	0 (0.00%)		
Glandular involvement, *n* (%):					0.374	41
No	4 (9.76%)	2 (12.5%)	2 (15.4%)	0 (0.00%)		
Yes	12 (29.3%)	3 (18.8%)	3 (23.1%)	6 (50.0%)		
Unknown	25 (61.0%)	11 (68.8%)	8 (61.5%)	6 (50.0%)		
Quadrants affected, *n* (%):					0.940	27
0	1 (3.70%)	1 (9.09%)	0 (0.00%)	0 (0.00%)		
1	3 (11.1%)	1 (9.09%)	1 (11.1%)	1 (14.3%)		
2	5 (18.5%)	1 (9.09%)	3 (33.3%)	1 (14.3%)		
3	5 (18.5%)	2 (18.2%)	2 (22.2%)	1 (14.3%)		
4	13 (48.1%)	6 (54.5%)	3 (33.3%)	4 (57.1%)		
Circumference *, Median (Q1; Q3)	5.34 (4.40; 6.28)	4.71 (3.69; 5.50)	5.50 (5.11; 6.28)	5.50 (4.01; 6.36)	0.182	26
Thickness, Median (Q1; Q3)	1.00 (0.88; 1.15)	1.05 (0.90; 1.20)	1.00 (0.88; 1.06)	1.07 (0.89; 1.15)	0.651	23
Volume LLETZ Carcopino **, *n* (%)	1.81 (1.25; 2.26)	1.14 (0.96; 1.52)	1.93 (1.81; 2.26)	5.06 (3.97; 6.14)	0.017	13
Volume LLETZ Phadnis ***, Median (Q1; Q3)	0.58 (0.40; 0.72)	0.36 (0.31; 0.48)	0.61 (0.58; 0.72)	1.61 (1.26; 1.95)	**0.017**	13
Length, Median (Q1; Q3)	1.10 (0.78; 1.50)	0.60 (0.50; 0.75)	1.20 (1.00; 1.22)	2.10 (2.00; 3.00)	**<0.001**	20
Endocervical canal extension *, *n* (%)	10 (24.4%)	0 (0.00%)	2 (15.4%)	8 (66.7%)	**<0.001**	41
**Follow-up**						
LOG (HIV Viral Load at LLETZ), Median (Q1; Q3)	0.00 (0.00; 4.60)	0.00 (0.00; 0.00)	4.60 (0.00; 8.30)	2.30 (0.00; 4.60)	**0.020**	41
HIV Viral load at LLETZ, Median (Q1; Q3)	0.00 (0.00; 99.0)	0.00 (0.00; 0.00)	99.0 (0.00; 4039)	49.5 (0.00; 99.0)	**0.020**	41
LOG (CD4 at LLETZ), Median (Q1; Q3)	0.00 (0.00; 5.85)	0.00 (0.00; 0.00)	3.21 (0.00; 5.75)	0.00 (0.00; 6.18)	0.141	39
LOG (CD4 (%) at LLETZ), Median (Q1; Q3)	0.00 (0.00; 2.82)	0.00 (0.00; 0.00)	1.96 (0.00; 2.88)	1.32 (0.00; 3.33)	0.078	40
Follow-up (months), Median (Q1; Q3)	11.9 (4.96; 50.5)	11.0 (6.12; 26.1)	7.26 (3.88; 72.1)	21.8 (7.06; 53.8)	0.655	41

Abbreviations: HR-HPV, high-risk human papillomavirus; RLU, relative light units; ASC-US, atypical squamous cells of undetermined significance; HSIL, high-grade squamous intraepithelial lesion; HIV, Human Immunodeficiency Virus; CD4, Cluster of Differentiation 4 T-lymphocyte count (CD4+ T cells). Surgical specimen parameters were defined according to the 2011 colposcopic terminology of the International Federation of Cervical Pathology and Colposcopy (IFCPC) [54]. LOG *values* correspond to natural logarithms (ln). To recover the original clinical values, use the $e^{x}$ button on a calculator (*Original Value =* $e^{x}$ LOG *value*). HIV RNA was categorised according to HHS guidance as: virologic suppression (<20 copies/mL), low-level viremia (20–199 copies/mL) and virologic failure (persistent HIV RNA ≥ 200 copies/mL despite antiretroviral therapy) [69]. CD4 counts were interpreted according to CDC immunologic staging: stage 1, ≥500 cells/mm^3^ or ≥26%; stage 2, 200–499 cells/mm^3^ or 14–25%; and stage 3, <200 cells/mm^3^ or <14% or any AIDS-defining condition [67,68]. Bold values indicate statistically significant differences between groups. * Only in immunocompetent patients. ** *Carcopino* formula: $\frac{1}{2}\times \frac{4}{3}\times \pi \times Lenght\times \frac{Circumference}{2\pi }\times Thickness$ [73]. *** *Phadnis* formula: $\frac{1}{2}\times \frac{4}{3}\times \pi \times \frac{transverse\;diameter}{2}\times \frac{longitudinal\;diameter}{2}\times depth$ [72].

## Appendix B

**Table A2:** Cox regression analysis of associated factors of residual/recurrent cervical intraepithelial neoplasia grade 2–3 (CIN 2–3), according to excision type, in immunosuppressed women treated with loop electrosurgical excision procedure (LLETZ) at Bellvitge University Hospital, Barcelona, Spain (1996–2016).

	Total	No Residual/Recurrent CIN 2–3	Residual/Recurrent CIN 2–3	HR	*p* Value	*n*
	*n* = 41	*n* = 27	*n* = 14			
Type of treatment, *n* (%):						41
Excision type 1, *n* (%)	16 (39.0%)	11 (40.7%)	5 (35.7%)	ref.	ref.	
Excision type 2, *n* (%)	13 (31.7%)	9 (33.3%)	4 (28.6%)	0.53 (0.06; 4.52)	0.489	
Excision type 3, *n* (%)	12 (29.3%)	7 (25.9%)	5 (35.7%)	0.72 (0.13; 4.11)	0.675	
Age, Median [Q1; Q3]	37.5 (31.2; 45.1)	37.5 (31.1; 46.3)	37.9 (34.8; 43.8)	1.00 (0.95; 1.04)	0.857	41
Categorized Age, *n* (%):						41
<35	17 (41.5%)	12 (44.4%)	5 (35.7%)	Ref.	Ref.	
35+	24 (58.5%)	15 (55.6%)	9 (64.3%)	1.40 (0.47; 4.21)	0.549	
First HR-HPV post-LLETZ, *n* (%):						40
Negative	10 (25.0%)	9 (33.3%)	1 (7.69%)	Ref.	Ref.	
Positive	19 (47.5%)	9 (33.3%)	10 (76.9%)	6.20 (0.79; 48.6)	0.082	
Not performed	11 (27.5%)	9 (33.3%)	2 (15.4%)	1.52 (0.14; 16.9)	0.733	
Thickness, Median [Q1; Q3]	1.00 (0.88; 1.15)	1.05 (0.92; 1.15)	0.85 (0.75; 1.00)	0.03 (0.00; 9.46)	0.239	23
Volume LLETZ Carcopino **, *n* (%)	1.81 (1.25; 2.26)	1.84 (1.43; 2.39)	1.22 (1.12; 1.32)	0.26 (0.02; 3.40)	0.305	13
Volume LLETZ Phadnis ***, Median (Q1; Q3)	0.58 (0.40; 0.72)	0.59 (0.46; 0.76)	0.39 (0.36; 0.42)	0.01 (0.00; 46.9)	0.305	13
LOG (Viral load at LLETZ), Median (Q1; Q3)	0.00 (0.00; 4.60)	0.00 (0.00; 4.60)	0.00 (0.00; 4.60)	1.07 (0.93; 1.22)	0.361	41
Viral load at LLETZ, Median (Q1; Q3)	0.00 (0.00; 99.0)	0.00 (0.00; 99.0)	0.00 (0.00; 99.0)	1.00 (1.00; 1.00)	**0.018**	41
LOG (CD4 at LLETZ), Median (Q1; Q3)	0.00 (0.00; 5.85)	0.00 (0.00; 5.86)	0.00 (0.00; 3.33)	0.95 (0.78; 1.15)	0.575	39
LOG (CD4 (%) at LLETZ), Median (Q1; Q3)	0.00 (0.00; 2.82)	0.00 (0.00; 2.89)	0.00 (0.00; 2.48)	0.93 (0.66; 1.32)	0.695	40
Margin status, *n* (%):						41
Negative margins	17 (41.5%)	12 (44.4%)	5 (35.7%)	Ref.	Ref.	
Ecto+/−, Endo+/−, All, Depth	19 (46.3%)	10 (37.0%)	9 (64.3%)	2.90 (0.95; 8.88)	0.062	
Indeterminate	5 (12.2%)	5 (18.5%)	0 (0.00%)	0.00 (0.00;)	0.999	
Margin status, *n* (%):						41
Negative margins	17 (41.5%)	12 (44.4%)	5 (35.7%)	Ref.	Ref.	
Ecto+/endocervical−	8 (19.5%)	5 (18.5%)	3 (21.4%)	1.72 (0.41; 7.25)	0.457	
Ecto−/endocervical+	5 (12.2%)	2 (7.41%)	3 (21.4%)	6.06 (1.27; 28.9)	**0.024**	
Ecto+/endocervical+	3 (7.32%)	1 (3.70%)	2 (14.3%)	14.0 (2.11; 92.4)	**0.006**	
All margins	3 (7.32%)	2 (7.41%)	1 (7.14%)	2.10 (0.23; 19.4)	0.514	
Indeterminate	5 (12.2%)	5 (18.5%)	0 (0.00%)	0.00 (0.00;)	0.998	
Quadrants affected, *n* (%):						27
0–1	4 (14.8%)	4 (23.5%)	0 (0.00%)	Ref.	Ref.	
2	5 (18.5%)	4 (23.5%)	1 (10.0%)	114,245,421 (0.00;)	0.999	
3	5 (18.5%)	2 (11.8%)	3 (30.0%)	728,679,448 (0.00;)	0.999	
4	13 (48.1%)	7 (41.2%)	6 (60.0%)	325,950,572 (0.00;)	0.999	
First HPV RLU post-LLETZ, *n* (%):						41
0–100	17 (41.5%)	13 (48.1%)	4 (28.6%)	Ref.	Ref.	
>100	7 (17.1%)	2 (7.41%)	5 (35.7%)	5.54 (1.45; 21.1)	**0.012**	
Unknown	17 (41.5%)	12 (44.4%)	5 (35.7%)	1.12 (0.30; 4.20)	0.862	

Abbreviations: HR, hazard ratio; HR-HPV, high-risk human papillomavirus; RLU, relative light units. Immunosuppressed women include those living with HIV or those with solid-organ transplantation/other causes of chronic immunosuppression. Residual/recurrent disease: CIN 2–3 lesions diagnosed after LLETZ excisional treatment, classified as residual if occurring within 12 months of follow-up, or recurrent if occurring after 12 months. Surgical specimen parameters were defined according to the 2011 International Federation of Cervical Pathology and Colposcopy (IFCPC) terminology [54]. LOG *values* correspond to natural logarithms (ln). To recover the original clinical values, use the $e^{x}$ button on a calculator (*Original Value =* $e^{x}$ LOG *value*). HIV RNA was categorised according to HHS guidance as: virologic suppression (<20 copies/mL), low-level viremia (20–199 copies/mL) and virologic failure (persistent HIV RNA ≥ 200 copies/mL despite antiretroviral therapy) [69]. CD4 counts were interpreted according to CDC immunologic staging: stage 1, ≥500 cells/mm^3^ or ≥26%; stage 2, 200–499 cells/mm^3^ or 14–25%; and stage 3, <200 cells/mm^3^ or <14% or any AIDS-defining condition [67,68]. Bold values indicate statistically significant differences between groups. ** *Carcopino* formula: $\frac{1}{2}\times \frac{4}{3}\times \pi \times Lenght\times \frac{Circumference}{2\pi }\times Thickness$ [73]. *** *Phadnis* formula: $\frac{1}{2}\times \frac{4}{3}\times \pi \times \frac{transverse\;diameter}{2}\times \frac{longitudinal\;diameter}{2}\times depth$ [72].
